# Prevalence of extended-spectrum *β*-lactamase-producing *Escherichia coli* in various animal-derived food products

**DOI:** 10.3389/fvets.2026.1871688

**Published:** 2026-06-12

**Authors:** Toray Ilbars Boz, Ali Anil Suleymanoglu, Ali Aydin

**Affiliations:** Department of Food Hygiene and Technology, Faculty of Veterinary Medicine, Istanbul University-Cerrahpasa, Istanbul, Türkiye

**Keywords:** carbapenem resistance, *Escherichia coli*, extended spectrum *β*-lactamase, mobilized colistin resistance, public health

## Abstract

Various strains of *Escherichia coli* are significant foodborne pathogens that can threaten public health through contaminated food. In this study, a total of 217 animal-derived food products, including raw turkey meat, raw beef, fresh cheese, and raw milk, were collected from markets, butcher shops, dairies, and neighborhood markets in Istanbul during 2021, 2022, and 2023. *E. coli* isolates were detected in 13.4% of the samples using conventional microbiological methods, and identification was confirmed by PCR targeting the *E. coli*-specific 16S rRNA gene region. The proportions of *E. coli* isolated were: 26.1% from raw turkey meat, 15.4% from raw beef, 8% from raw milk, and 0% from fresh cheese. The isolates were further analyzed by PCR for the presence of *stx1*, *stx2*, and *rfbO157* genes; none of the isolates carried these virulence genes. Antibiotic susceptibility testing by the disk diffusion method revealed resistance rates of 93.1% to ampicillin; 62% to ceftazidime, cefotaxime, amoxicillin/clavulanate, and chloramphenicol; and 55.1% to meropenem. Multidrug-resistant *E. coli* isolates accounted for 75.8% of the total, and 51.7% were identified as extended-spectrum *β*-lactamase producers by the double-disk synergy test. PCR analysis showed that 82.7% of isolates carried the *bla*_TEM_ gene. The mPCR method was used to detect *bla*_CTX-M_ and yielded positive results in 6.8% (2/29) of group 1, 65% (19/29) of group 2, 10% (3/29) of group 8 and 25/26, and 13.7% (4/29) of group 9. No carbapenemase or mobilized colistin resistance genes were detected. In this study, the biofilm-forming capacity of *E. coli* isolates was measured using a microplate method; notably, 75% of the raw milk-derived isolates formed biofilms in Brain Heart Infusion Broth. The results of this study indicate that antibiotic resistance rates in foodborne *E. coli* isolates are at alarming levels for public health, and that immediate preventive measures should be taken.

## 1 Introduction

*Escherichia coli* (*E. coli*) is a commensal bacterium commonly found in the intestinal microbiota of warm-blooded animals and humans. It is also recognized as a significant zoonotic pathogen (1). Moreover, *E. coli* is used as an indicator of fecal contamination in food products (2), and specific strains have been documented to cause severe infections due to their virulence factors.

Among foodborne pathogenic *E. coli* strains, Enterohaemorrhagic *E. coli* (EHEC) has recently garnered significant scholarly and public health attention. STEC (Shiga Toxin-Producing *Escherichia coli*) and EHEC are groups of pathogenic bacteria that cause serious foodborne illnesses, and they are related in a “supergroup-subgroup” relationship. STEC is a broad umbrella term encompassing all *E. coli* strains capable of producing Shiga toxin (Stx1 and/or Stx2); EHEC is a specific subset of this group that is clinically the most dangerous and causes severe conditions in humans, such as hemorrhagic colitis and hemolytic uremic syndrome (3). The primary reservoir for EHEC is the bovine gastrointestinal tract, and initial outbreaks are predominantly associated with the ingestion of undercooked hamburgers. Since then, EHEC outbreaks have been linked to a diverse array of food products. Transmission occurs via multiple pathways, including contaminated tap water and direct contact with farm environments, facilitated by the remarkably low infectious dose—estimated at fewer than 100 bacterial cells (4). In 2022, 29 European Union and European Economic Area member states reported 8,565 cases of (STEC) infection. The overall notification rate reached 2.5 cases per 100,000 inhabitants, surpassing pre-pandemic levels and reflecting a 25% increase from 2021. Correspondingly, the number of confirmed HUS cases also increased in 2022. Of the 568 reported HUS cases, the majority occurred among the youngest cohorts—60% in children aged 0–4 years and 24% in those aged 5–14 years. Nonetheless, mortality rates were higher in older populations (5). The infectious dose of EHEC strains is extremely low, and even a small number of EHEC bacteria are sufficient to cause serious outbreaks. For this reason, it is one of the *E. coli* types that requires the most attention in food hygiene and public health efforts (6). Additionally, due to its severe pathological effects, such as hemolytic uremic syndrome, it is classified as a zero-tolerance pathogen (7).

Antimicrobial resistance (AMR) and multidrug resistance (MDR) pose significant threats to public health. Globally, approximately 700,000 people die each year from infections associated with AMR, and this figure is projected to reach 10 million annually by 2050 in the absence of effective interventions (8). In the United States, an estimated 2.6 million people acquire infections attributable to antibiotic-resistant bacteria each year, with an average mortality rate of 1.7% (9). The widespread and inappropriate use of antibiotics in livestock production and human healthcare significantly contributes to the unregulated rise of antimicrobial resistance (10). Current research indicates the presence of multidrug-resistant *E. coli* strains in food animals and in the food supply (11–13).

Extended-Spectrum Beta-Lactamases (ESBLs) are enzymes produced by bacteria that inactivate a broad group of beta-lactam antibiotics—such as penicillins, cephalosporins (1st, 2nd, 3rd, and 4th generations), and monobactams (aztreonam)—by hydrolyzing them (14). In the context of antimicrobial resistance, ESBL-producing and carbapenem-resistant *Enterobacterales* pose a significant threat to global health, according to the World Health Organization (15). Within *Enterobacterales*, *E. coli* is a predominant contributor to the production of extended-spectrum *β*-lactamases (ESBLs) and to the development of carbapenem resistance. It demonstrates remarkable efficiency in disseminating these resistance traits via plasmids, which are mobile genetic elements. Although carbapenems are effective against bacteria resistant to cephalosporins and penicillins, their increased use has facilitated the emergence of carbapenem-resistant strains. As a result, the utilization of colistin—a last-resort antibiotic—has increased. The renewed dependence on colistin, despite its well-documented nephrotoxic and neurotoxic effects, highlights the severity of the current antimicrobial resistance crisis (13). Current research indicates the presence of foodborne ESBL-producing, carbapenem-resistant, or colistin-resistant *E. coli* in food animals. In this context, studies on the spread of beta-lactam, ESBL, carbapenem, and colistin resistance through food have been conducted across various food products and regions (11, 16–20).

Biofilms are intricate structures that bolster bacterial persistence across various environments, with *E. coli* among the most prominent Gram-negative species in this context. Biofilms pose a significant public health concern, as they not only exhibit increased resistance to disinfectants but also adversely affect the food industry and contribute to the escalating prevalence of antibiotic resistance (21). The formation of biofilms markedly decreases the efficacy of antibiotic treatments. Although antibiotic resistance is often assessed using the minimum inhibitory concentration (MIC) in planktonic cell cultures, biofilm development significantly alters these values, thereby reducing therapeutic effectiveness (22).

This study was conducted in Türkiye, where resources for advanced molecular analyses are limited due to economic constraints. Additionally, import issues in Türkiye have also limited the use of next-generation techniques. Because our country serves as a transit point and may host many different types of plasmids, selecting target genes is difficult. Although there are few comprehensive publications on ESBL-producing *E. coli* in the context of food safety in Türkiye (17, 23, 24) data on turkey meat are particularly limited. Concurrent studies on this topic across different food products and the measurement of biofilm formation have been conducted in only a small number of studies. The objective of this study is to: (a) determine the prevalence of *E. coli* in raw beef, raw turkey meat, raw milk, and fresh cheese obtained from Istanbul; (b) investigate whether the isolated *E. coli* strains are EHEC; (c) determine the antibiotic resistance profiles of *E. coli* isolates; (d) examine the presence of ESBL, carbapenem, and mobilized colistin resistance genes utilizing PCR; (e) conduct a investigation into ESBL-producing *E. coli* in various animal-derived food products; and (f) assess biofilm formation by 29 isolated ESBL *E. coli* isolates in three distinct media in a major metropolis such as Istanbul.

## 2 Materials and methods

### 2.1 Sampling

A total of 217 food samples were collected, including raw turkey samples (*n* = 65) in 2021, raw red meat samples (*n* = 52) in 2022, raw milk (cow) samples (*n* = 50) in 2023, and fresh cheese samples (*n* = 50) in 2023. These samples were obtained from various markets, butchers, dairies, and neighborhood markets in Istanbul. Subsequently, the samples were transported to the Food Hygiene and Technology Department Laboratory at Istanbul University-Cerrahpasa under cold storage conditions (+4 °C) and analyzed immediately.

### 2.2 Isolation and identification of *Escherichia coli* by conventional methods

The ISO 16649-2:2001 (25) standard was used to isolate *E. coli*. Subsequently, 25 g of beef, turkey, or cheese samples were weighed and placed into stomacher bags. Additionally, 25 mL of raw milk samples were utilized; to these, 225 mL of Buffered Peptone Water (BPW; Oxoid CM 0509, Basingstoke, United Kingdom) was added. The mixture was then homogenized in a stomacher (Interscience, France) for 2 min. Following homogenization, the specimen was inoculated onto Tryptone Bile X Glucuronide Agar (Oxoid CM 0945), streaked with a Drigalski rod, and incubated at 44 °C for 24 h. Suspected colonies were then transferred to Eosin Methylene Blue Agar (EMB; Oxoid 0069B) to confirm microbiological identity and incubated at 37 °C for 24 h. *E. coli* ATCC 25922 has been used as a reference strain for the verification of *E. coli*. *S. infantis* ATCC 51741 was used as the negative control strain for isolation and identification.

These representative colonies were aseptically picked using a sterile inoculation loop and transferred onto Tryptic Soy Agar (TSA; Oxoid CM0131) plates using the streak plate method to obtain isolated, pure colonies and ensure phenotypic homogeneity. The cultures were on TSA incubated at 37 °C for 24 h. Isolates confirmed to be pure were then transferred to Tryptone Soya Broth (TSB; Oxoid CM129) supplemented with 20% glycerol (Sigma-Aldrich, G5516, Germany) and stored at −20 °C for future reference.

### 2.3 Verification of *Escherichia coli* isolates by PCR

#### 2.3.1 DNA extraction

DNA extraction for subsequent molecular genetic analyses was performed using the method described by Liu et al. (26).

#### 2.3.2 Confirmation of *Escherichia coli* isolates by PCR (16S rRNA)

The DNA extracted from the suspected *E. coli* isolates was subjected to PCR analysis using ECO 1–2 primers targeting the 16S rRNA gene (Table 1). Each reaction mixture contained the following components: 2.5 μL of DNA template, 2.5 μL of 10X KCl (potassium chloride)reaction buffer (Thermo Fisher Scientific), 2.5 μL of a dNTP (deoxynucleotide triphosphate) mixture (dATP, dTTP, dGTP, and dCTP) (R0192; Thermo Fisher Scientific), 1.5 μL of MgCl₂ (magnesium chloride) (25 mM; Thermo), 0.5 μL of forward primer (10 μM), 0.5 μL of reverse primer (10 μM), 1 U of Taq DNA polymerase (Thermo Fisher EP0404; Thermo Fisher Scientific, Waltham, MA, United States), and nuclease-free distilled water to a final volume of 25 μL. The PCR cycling protocol for ECO 1–2 consisted of an initial denaturation at 95 °C for 5 min (min), followed by 30 cycles of denaturation at 95 °C for 30 s (sec), annealing at 55 °C for 45 s, and extension at 72 °C for 45 s. A final extension at 72 °C for 7 min was performed (27). Electrophoretic visualization of the PCR products was performed on agarose gels stained with SafeView^™^ Classic (ABM, Richmond, BC, Canada) and imaged using the Infinity Gel Imaging System (Vilber Lourmat, Marne-la-Vallée, France). *E. coli* ATCC 25922 has been used as a reference strain for the verification of *E. coli*. *S. infantis* ATCC 51741 was used as the negative control strain for isolation and identification.

**Table 1:** Primers and target genes for the PCR detection of virulence genes, ESBL genes, carbapenem resistance genes, and mobilized colistin resistance genes.

Amplicon	Primer sequence (5′—›3′)	Size (Bp)	Reference
*bla*_SHV_	F: CTTTATCGGCCCTCACTCAA R: AGGTGCTCATCATGGGAAAG	237	(33)
*bla*_TEM_	F: CGCCGCATACACTATTCTCAGAATGA R: ACGCTCACCGGCTCCAGATTTAT	445	(33)
*bla*_CTX-M_	F: ATGTGCAGYACCAGTAARGTKATGGC R: TGGGTRAARTARGTSACCAGAAYCAGCGG	593	(33)
*bla*_OXA_	F: ACACAATACATATCAACTTCGC R: AGTGTGTTTAGAATGGTGATC	813	(33)
*bla*_OXA-48_	F: TTGGTGGCATCGATTATCGG R: GAGCACTTCTTTTGTGATGGC	744	(35)
*bla*_NDM_	F: TGGCAGCACACTTCCTATC R: AGATTGCCGAGCGACTTG	488	(35)
*bla*_KPC_	F: CTGTCTTGTCTCTCATGGCC R: CCTCGCTGTRCTTGTCATCC	796	(35)
*bla*_VIM_	F: AGTGGTGAGTATCCGACAG R: TCAATCTCCGCGAGAAG	212	(35)
*16S rRNA*	F: GACCTCGGTTTAGTTCACAGA R: CACACGCTGACGCTGACCA	585	(27)
*stx1*	F: CACAATCAGGCGTCGCCAGCGCACTTGCT R: TGTTGCAGGGATCAGTGGTACGGGGATGC	606	(28)
*stx2*	F: CCACATCGGTGTCTGTTATTAACCACACC R: GCAGAACTGCTCTGGATGCATCTCTGGTC	372	(28)
*rfbO157*	F: AAGATTGCGCTGAAGCCTTTG R: CATTGGCATCGTGTGGACAG	497	(28)
*mcr-1*	F: AGTCCGTTTGTTCTTGTGGC R: AGATCCTTGGTCTCGGCTTG	320	(35)
*mcr-2*	F: CAAGTGTGTTGGTCGCAGTT R: TCTAGCCCGACAAGCATACC	715	(35)
*mcr-3*	F: AAATAAAAATTGTTCCGCTTATG R: AATGGAGATCCCCGTTTTT	929	(35)
*mcr-4*	F: TCACTTTCATCACTGCGTTG R: TTGGTCCATGACTACCAATG	1,116	(35)
*mcr-5*	F: ATGCGGTTGTCTGCATTTATC R: TCATTGTGGTTGTCCTTTTCTG	1,644	(35)
*bla*_CTX-M_ Group1	F: GCGTGATACCACTTCACCTC R: TGAAGTAAGTGACCAGAATC	260	(34)
*bla*_CTX-M_ Group2	F: TGATACCACCACGCCGCTC R: TATTGCATCAGAAACCGTGGG	341	(34)
*bla*_CTX-M_ Group 8 and 25/26	F: CAATCTGACGTTGGGCAATG R: ATAACCGTCGGTGACAATT	207	(34)
*bla*_CTX-M_ Group9	F: ATCAAGCCTGCCGATCTGGTTA R: GTAAGCTGACGCAACGTCTGC	293	(34)

#### 2.3.3 Detection of virulence genes in *Escherichia coli* strains

The virulence genes (*stx1*, *stx2*, and *rfbO157*) of *E. coli* (Table 1) were analyzed by monoplex PCR. Each reaction mixture contained the following components: 2.5 μL of DNA template; 2.5 μL of 10X KCl reaction buffer; 2.5 μL of dNTP mixture; 1.5 μL of (MgCl_₂_; 25 mM; Thermo); 0.5 μL of forward primer specific to each virulence gene (10 μM); 0.5 μL of reverse primer specific to each virulence gene (10 μM); 1 unit of Taq DNA polymerase; and nuclease-free deionized water (dH₂O) to a final volume of 25 μL. Thermal cycling for detecting the virulence genes included an initial denaturation at 95 °C for 5 min, followed by 30 amplification cycles consisting of denaturation at 94 °C for 30 s, annealing at 66 °C for 45 s for *rfbO157* and at 58 °C for 45 s for *stx1* and *stx2*, with extension at 72 °C for 1 min. The cycle concluded with a final extension at 72 °C for 10 min (28). The PCR products were visualized on agarose gels stained with SafeView™ using the Infinity Gel Imaging System. *E. coli* ATCC 43895 was used as the reference strain for *E. coli* O157: H7.

### 2.4 Antibiotic susceptibility tests in *Escherichia coli* isolates

Initially, the isolates were cultivated on TSA and then meticulously transferred to the surface of Mueller-Hinton Agar (MHA; Oxoid, CM0337) using a Drigalski spatula. The bacterial suspension was prepared in physiological saline in glass tubes and standardized to the McFarland 0.5 standard using a sterile swab (29). Subsequently, antibiotic disks were systematically placed on MHA plates in accordance with EUCAST (30) and CLSI (31) guidelines for *E. coli*. The following antibiotic disks were used: ampicillin (AMP; Oxoid, CT0003B, 10 μg), amoxicillin-clavulanic acid (AMC; Oxoid, CT0223B, 30 μg), cefotaxime (CTX; Oxoid, CT0166B, 30 μg), ceftazidime (CAZ; Oxoid, CT0412B, 30 μg), chloramphenicol (C; Oxoid, CT0013B, 30 μg), meropenem (MEM; Oxoid, CT0774B, 10 μg), trimethoprim-sulfamethoxazole (SXT; Oxoid, CT0025B, 1.25 μg–23.5 μg), ciprofloxacin (CIP; Oxoid, CT0425B, 5 μg), and tetracycline (TE; Oxoid, CT0054B, 30 μg). The Petri dishes were incubated at 37 °C for 18 ± 2 h. *E. coli* ATCC 25922 was used as the negative control strain in the disk diffusion test. The antibiotics examined in this study were selected based on the “WHO List of Bacterial Priority Pathogens” (15), with a focus on those that may be associated with these resistance mechanisms.

#### 2.4.1 Phenotypic determination for ESBL in *Escherichia coli* isolates

The isolates were incubated on TSA, suspended in physiological saline in glass tubes, and adjusted to the McFarland 0.5 turbidity standard. The suspension was then spread evenly over MHA using a Drigalski spatula. In the double-disk synergy test, an amoxicillin-clavulanic acid disk (Oxoid, CT0223B, 30 μg) was placed at the center of the plate, with cefotaxime (Oxoid, CT0166B, 30 μg) and ceftazidime (Oxoid, CT0412B, 30 μg) disks placed 20 mm apart on either side. The plates were incubated at 37 °C for 24 h. According to EUCAST (32) guidelines, a result is considered positive for ESBL production if the inhibition zone of either cephalosporin disk enlarges toward the clavulanic acid disk, indicating synergy.

#### 2.4.2 Genotypic determination of antibiotic resistance genes in *Escherichia coli* isolates

Multiplex PCR was used to detect ESBL genes (*bla*_TEM_, *bla*_SHV_, *bla*_CTX-M_, and *bla*_OXA_) (Table 1). The reaction mixtures consisted of 2.5 μL of DNA, 2.5 μL of 10X reaction buffer KCl (Thermo), 2.5 μL of dNTP mixture, 1.5 μL of MgCl_2_ (25 mM) (Thermo), 0.5 μL of forward primer for each target gene (10 μM), 0.5 μL of reverse primer for each target gene (10 μM), 1 U of Taq DNA polymerase, and nuclease-free water combined with 25 μL of master mix. The PCR conditions for multiplex ESBL detection were as follows: an initial denaturation at 95 °C for 15 min, followed by 30 cycles comprising denaturation at 94 °C for 30 s, annealing at 62 °C for 90 s, and extension at 62 °C for 60 s. A final extension was performed at 72 °C for 10 min (33). The amplification products were visualized on agarose gels stained with SafeView™ Classic stain (ABM, Richmond, BC, Canada) using the Infinity Gel Imaging System (Vilber Lourmat, Marne-la-Vallée, France). The PCR assay conducted to determine whether the isolates harbour *bla*_CTX-M_ subgroup (1–2, 8 and 25/26, 9) genes (34). The amplified PCR products were subjected to electrophoresis on a 1.5% agarose gel using a 5 μL SafeView (Abm, Richmond, Canada) (Table 1).

The monoplex PCR methodology was used to detect carbapenem resistance genes, specifically *bla*_OXA-48_, *bla*_NDM_, *bla*_VIM_, and *bla*_KPC_ (Table 1). For each sample, 2.5 μL of DNA, 2.5 μL of 10X reaction buffer KCl (Thermo), 2.5 μL of dNTP mixture, 1.5 μL of MgCl_2_ (25 mM) (Thermo), 0.5 μL of each forward primer (10 μM), 0.5 μL of each reverse primer (10 μM), and 1 U of Taq DNA polymerase were combined with dH_2_O to reach a total volume of 25 μL. The PCR conditions for multiplex amplification of carbapenem resistance genes included an initial denaturation step at 94 °C for 5 min, followed by 30 cycles consisting of denaturation at 94 °C for 30 s, annealing at the specific melting temperature for 60 s, and extension at 72 °C for 60 s. A final extension at 72 °C for 10 min was also performed (35). *Klebsiella pneumoniae* NCTC 13443, harbouring the metallo-beta-lactamase NDM-1, was used as a reference strain.

The multiplex PCR technique was used to detect mobilized colistin resistance (*mcr*) genes, including *mcr*-1, *mcr*-2, *mcr*-3, *mcr*-4, and *mcr*-5, as detailed in Table 1. For the detection of *mcr* genes via multiplex PCR, the following conditions were used: an initial denaturation at 95 °C for 15 min, followed by 30 cycles of denaturation at 94 °C for 30 s, annealing at 58 °C for 90 s, and extension at 72 °C for 60 s. A final extension step was performed at 72 °C for 10 min (35). Visualization of the DNA bands in the agarose gels was performed using SafeView™ Classic stain within the Infinity Gel Imaging System. *E. coli* NCTC 13846 was used as the reference strain.

### 2.5 Biofilm formation in ESBL *Escherichia coli* isolates

Biofilm formation by isolated *E. coli* (*n* = 29) in 3 media was tested using the microplate method. Optical densities (OD) were measured at 595 nm using a microplate reader, and OD > 0.5 were considered indicative of biofilm formation (36). For this purpose, TSB, TSB containing 1% (*w*/*v*) sucrose, and Brain Heart Infusion Broth (BHI; Oxoid) were used in the microplate tests. Ninety-six-well F-bottom plates (Greiner BioOne Cell Star 655,180) were used, and absorbance at 595 nm was measured with a microplate reader (Epoch2, BioTek, Uited States). *Staphylococcus epidermidis* strain YT-169a was used as a positive control (37).

### 2.6 Statistical analysis

Correlation analysis was conducted using Pearson’s correlation test to assess relationships among biofilm measurements obtained in different culture media. Statistical analyses were performed using SPSS software (Version 29; IBM Corp., Armonk, NY, United States).

## 3 Results

### 3.1 Detection of *Escherichia coli* in chicken meat in various animal-derived food products samples in Istanbul

In this study, *E. coli* was detected in 13.4% (*n* = 29) of 217 food samples, including beef, turkey meat, raw milk, and fresh cheese. Prevalence varied by food type, with the highest incidence in raw turkey meat (26%; *n* = 17/65), followed by raw beef (15%; *n* = 8/52) and raw milk samples (12.5%; *n* = 4/50). Notably, no *E. coli* isolates were recovered from fresh cheese (*n* = 50).

In this study, PCR for *rfbO157*, *stx1* and *stx2* genes was used to determine whether the isolates were EHEC or STEC after *E. coli* detection; none of the isolates belonged to these pathogenic groups.

### 3.2 Phenotypic determination for antibiotic susceptibility in *E. coli* isolates

The antibiotic resistance profiles of the *E. coli* isolates identified in this study are summarized in Tables 2–4. A notably high rate of ampicillin resistance was observed, and resistance to cephalosporins, carbapenems, and fluoroquinolones was also detected at levels that raise significant public health concerns.

**Table 2:** Antibiotic resistance profiles of *E. coli* isolates according to EUCAST and CLSI guidelines.

Antibiotic group	Name of antibiotic	Distribution of *E. coli* isolates according to EUCAST (30) and CLSI (31)		
		Raw beef	Raw Türkiye	Raw milk
		R (%)	R (%)	R (%)
		(*n* = 8)	(*n* = *17*)	(*n* = *4*)
Cephalosporins	Cefotaxime 30 μg	62.5% (*n* = *5*)	58.8% (*n* = 10)	75% (*n* = *3*)
	Ceftazidime 30 μg	62.5% (*n* = *5*)	58.8% (*n* = 10)	75% (*n* = *3*)
Carbapenems	Meropenem 10 μg	62.5% (*n* = *5*)	52.9% (*n* = *9*)	50% (*n* = *2*)
Fluoroquinolones	Ciprofloxacin 5 μg	62.5% (*n* = 5)	58.9% (*n* = 10)	50% (*n* = 2)
Penicillin	Ampicillin 10 μg	100% (*n* = 8)	88.2% (*n* = *15*)	100% (*n* = 4)
	Amoxicillin clavulanic acid 30 μg	62.5% (*n* = *5*)	76.4% (*n* = *1*3)	75% (*n* = *3*)
Phenicol	Chloramphenicol 30 μg	62.5% (*n* = *5*)	76.4% (*n* = *1*3)	75% (*n* = *3*)
Sulfonamid	Trimethoprim-Sulfamethoxazole 25 μg	25% (*n* = 2)	29.4% (*n* = 5)	75% (*n* = 3)
Tetracyclines	Tetracycline 30 μg	50% (*n* = 4)	23.5% (*n* = 4)	25% (*n* = 1)

Resistant (“R”).

**Table 3:** Sources of *E. coli* isolates and rates of multidrug resistance.

Sample type	Number of samples collected	Sample year	Number of positive *E. coli* isolates	The antibiotic group with the highest resistance	Multiple antibiotic resistance rate
Raw beef	52	2022	8	Ampicillin	62.5%
Raw Türkiye	65	2021	17	Ampicillin	82%
Raw milk	50	2023	4	Ampicillin	75%

**Table 4:** The antibiotic resistance profile of *E. coli* isolates.

Sample type	Isolate ID	*bla*_CTX-M_, *bla*_TEM_	Antibiotic resistance profile
Raw beef	D101	*bla*_TEM+_ *bla*_CTX-M_	AMP, TE
Raw beef	D108	*bla*_TEM+_ *bla*_CTX-M_	AMP
Raw beef	D110-1	–	C, AMC, CAZ, CTX, MEM, AMP, SXT, TE, CIP
Raw beef	D110-2	*bla*_TEM_	C, AMC, CAZ, CTX, MEM, AMP, SXT, CIP, TE
Raw beef	D117-1	*bla*_CTX-M_ + *bla*_TEM_	C, AMC, CAZ, CTX, MEM, AMP, CIP
Raw beef	D120	*bla*_TEM_	C, AMC, CAZ, CTX, MEM, AMP, CIP
Raw beef	D123-1	*bla*_TEM+_ *bla*_CTX-M_	C, AMC, CAZ, CTX, MEM, AMP, CIP
Raw beef	D123-2	*bla*_TEM+_ *bla*_CTX-M_	AMP, TE
Raw Türkiye	H10	*bla*_TEM+_ *bla*_CTX-M_	C, AMC, CAZ, CTX, MEM, AMP, CIP
Raw Türkiye	H12	*bla*_CTX-M_ + *bla*_TEM_	AMP
Raw Türkiye	H13	*bla*_CTX-M_	CAZ, CTX, CIP
Raw Türkiye	H14	*bla*_TEM+_ *bla*_CTX-M_	C, AMC, AMP
Raw Türkiye	H15-1	*bla*_CTX-M_	C, AMC, CAZ, CTX, MEM, AMP
Raw Türkiye	H15-2	–	AMC, AMP
Raw Türkiye	H19	*bla*_TEM+_ *bla*_CTX-M_	AMP, TE
Raw Türkiye	H2	*bla*_TEM+_ *bla*_CTX-M_	C, AMC, CAZ, CTX, MEM, AMP, SXT, TE
Raw Türkiye	H20	*bla*_TEM_	C, AMC, CAZ, CTX, MEM, AMP, SXT, CIP
Raw Türkiye	H22	*bla*_TEM_	C, AMC, CAZ, CTX, MEM, AMP, SXT, CIP
Raw Türkiye	H23	–	C, AMC, CAZ, CTX, MEM, AMP
Raw Türkiye	H39	*bla*_TEM+_ *bla*_CTX-M_	C, AMC, AMP, CIP, TE
Raw Türkiye	H67	*bla*_TEM+_ *bla*_CTX-M_	C, AMC, AMP, CIP
Raw Türkiye	H68-1	*bla*_TEM+_ *bla*_CTX-M_	C, AMP, SXT, CIP,
Raw Türkiye	H68-2	*bla*_TEM+_ *bla*_CTX-M_	C, AMC, CAZ, CTX, MEM, AMP, SXT, CIP, TE
Raw Türkiye	H7	*bla*_TEM+_ *bla*_CTX-M_	C, AMC, CAZ, CTX, MEM, AMP, CIP
Raw Türkiye	H8	*bla*_TEM+_ *bla*_CTX-M_	C, AMC, CAZ, CTX, MEM
Raw milk	S189-1	*bla*_TEM_	C, AMC, CAZ, CTX, AMP, SXT, TE
Raw milk	S189-2	*bla*_TEM+_ *bla*_CTX-M_	C, AMC, CAZ, CTX, MEM, AMP, SXT, CIP
Raw milk	S205-1	*bla*_TEM+_ *bla*_CTX-M_	AMP
Raw milk	S205-2	*bla*_TEM+_ *bla*_CTX-M_	C, AMC, CAZ, CTX, MEM, AMP, SXT, CIP

AMP, Ampicillin; TE, Tetracycline; C, Chloramphenicol; AMC, Amoxicillin-clavulanic acid; CAZ, Ceftazidime; CTX, Cefotaxime; MEM, Meropenem; SXT, Trimethoprim-sulfamethoxazole; CIP, Ciprofloxacin.

### 3.3 Prevalence of ESBL-producing *E. coli* and carbapenem/colistin resistance

In this study, resistance to the third-generation cephalosporins (ceftazidime and cefotaxime) was detected in 62.5% of raw beef isolates, 58.8% of raw turkey meat isolates, and 75% of raw milk isolates. Phenotypic ESBL production was assessed using the double-disk synergy test, and 15 (51.7%) isolates were confirmed as ESBL producers. Molecular screening revealed *bla*_TEM_ in 24 *E. coli* isolates, and both *bla*_CTX-M_ and *bla*_TEM_ were detected in 19 isolates; *bla*_SHV_ and *bla*_OXA_ were not identified in any isolate. mPCR results for the *bla*_CTX-M_ gene indicate that some isolates harbour the resistance gene despite not exhibiting cefotaxime resistance. Therefore, the absence of resistance in the phenotype does not definitively indicate that the resistance gene is not present. To assess carbapenem resistance, meropenem resistance was first assessed by disk diffusion, yielding rates of 62.5% in raw beef, 52.9% in raw turkey meat, and 50% in raw milk isolates. None of the isolates carried carbapenem- or colistin-resistance genes.

The mPCR method was used to analyze *bla*_CTX-M_ presence and detected it in 6.8% (2/29) of group 1; 65% (19/29) of group 2; 10% (3/29) of group 8, and 25/26; and 13.7% (4/29) of group 9. Group 1 *bla*_CTX-M_ was detected in *E. coli* isolates from 5.8% raw turkey and 12.5% raw beef. Group 2 *bla*_CTX-M_ was detected in 70% (12/17) *E. coli* isolates from raw turkey; 50% (4/8) *E. coli* isolates from raw beef; and 75% (3/4) *E. coli* isolates from raw milk. Group 8 and 25/26 *bla*_CTX-M_ were detected in 11.5% (2/17) *E. coli* isolates from raw turkey, and 12.5% (1/8) *E. coli* isolates from raw beef. Group 9 *bla*_CTX-M_ was detected in 17.5% (3/17) *E. coli* isolates originating from raw turkey and 12.5% (1/8) *E. coli* isolate from raw beef. These results demonstrate the diversity of genes responsible for ESBL production and the challenges of investigating them.

### 3.4 Biofilm production

**Figure 1:**
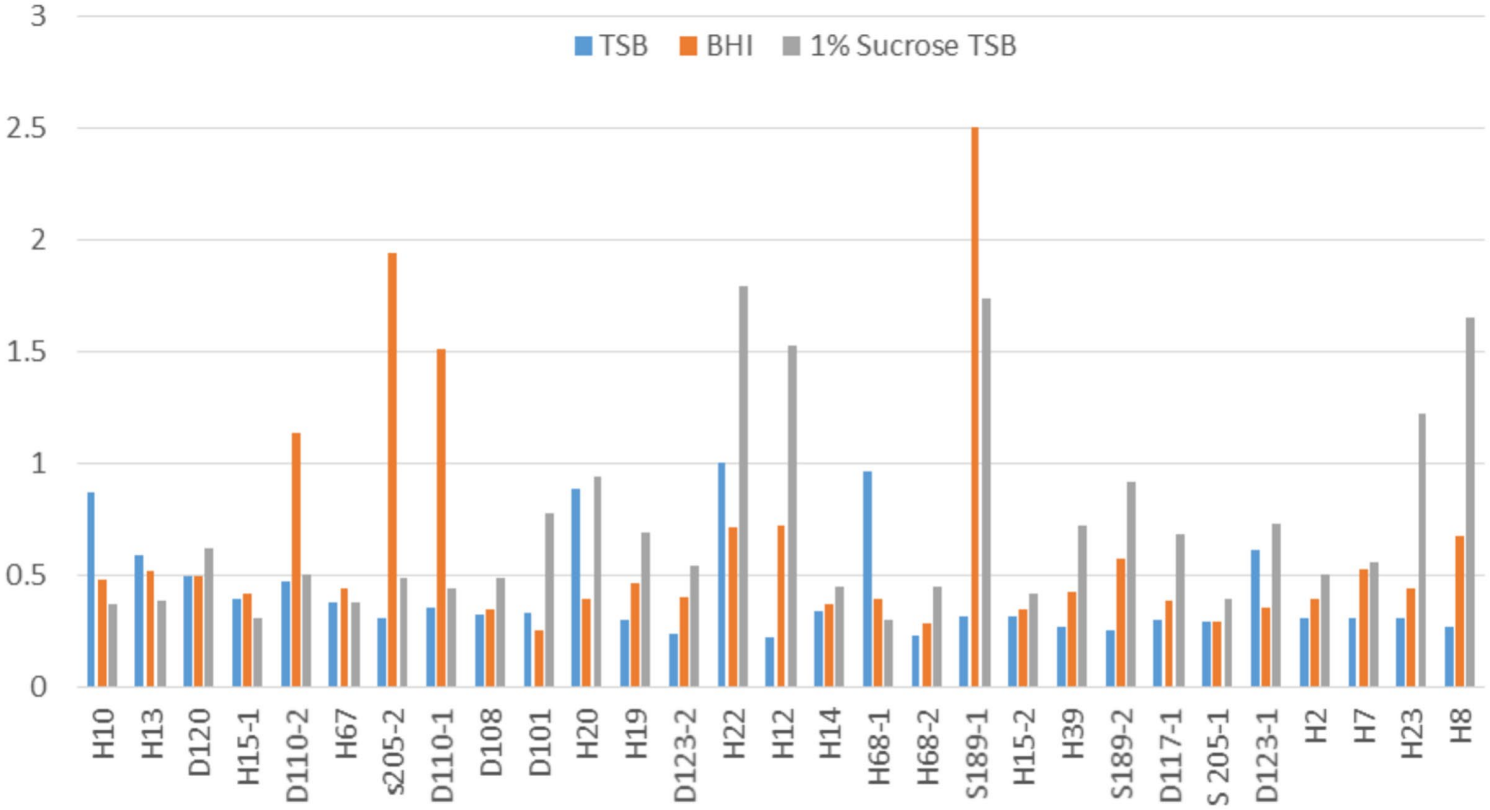
OD values at 595 nm of *E. coli* isolates in different media.

**Figure 2:**
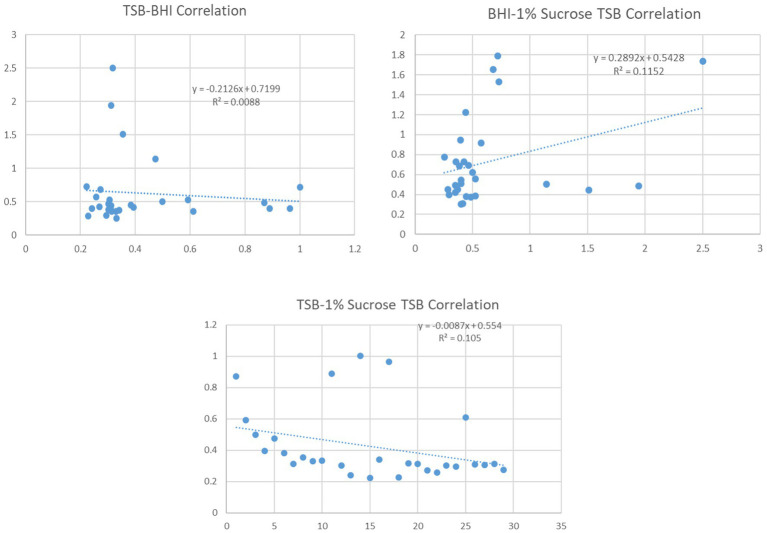
A correlation of biofilm formation among different broths.

This study assessed biofilm production across three distinct media: TSB, BHI, and 1% sucrose TSB- and identified 18 isolates that effectively produced biofilm in 1% sucrose TSB. Consequently, the biofilm formation capacity test conducted in these three media revealed that 16 isolates produced biofilm in 1% sucrose TSB, 11 in BHI, and 5 in TSB. The highest value recorded was 2.501 OD in BHI; notably, the isolate also produced substantial biofilm in 1% sucrose TSB (1.739). Overall, 29% of *E. coli* isolates from raw turkey meat produced biofilm in TSB. Specifically, 29% of *E. coli* isolates from raw turkey, 37.5% from raw beef, and 75% from raw milk produced biofilms in BHI. Additionally, 53% of *E. coli* isolates from turkey, 62.5% from raw beef, and 50% from raw milk formed biofilms in 1% sucrose TSB, as shown in Figure 1, 2.

The vertical column contains OD values at 595 nm, and the horizontal column contains sample numbers.

Figure 2 shows that when the biofilm-forming capacities of *E. coli* isolates were compared across three culture media, no significant correlation was observed (*p* > 0.05).

## 4 Discussion

Foodborne *E. coli* poses a significant threat to public health, serving as both a pathogen capable of causing disease and a reservoir of antibiotic resistance mechanisms that can be transferred among bacterial populations. Its prevalence has been documented across various food products, underscoring its widespread distribution and emphasizing the need for ongoing surveillance and control strategies within the food supply chain (16, 17, 38).

Zao et al. (39) reported *E. coli* in 11.9% of turkey meat samples, whereas Cook et al. (39, 40) reported a markedly higher prevalence of 95%, and Aworh et al. (41) reported 56.5%. These findings highlight the substantial variability in the prevalence of *E. coli* in poultry products across countries and time periods. Such variation may be attributable not only to geographical and temporal factors but also to differing methodological approaches. Specifically, studies that rely solely on conventional microbiological techniques tend to report higher prevalence rates. Conversely, the current study employed a more rigorous methodology, using both selective and differential media (chromogenic agar and EMB agar) and molecular confirmation targeting the *E. coli*-specific gene region, which likely contributed to the comparatively lower prevalence observed. Furthermore, unlike most prior research that focused exclusively on poultry, this investigation encompassed raw beef, raw milk, and fresh cheese, thereby providing a broader perspective on the distribution of *E. coli* across various animal-derived food products (42, 43).

Drugea et al. (44) reported *E. coli* in 22.5% of mastitic cow milk samples, Kurekci et al. (23) detected it in 22% of bulk tank milk, and Ombarak et al. (18) reported a considerably higher prevalence of 77% in raw milk obtained from local markets, farmer vendors, and supermarkets. In contrast, *E. coli* was isolated at 8% from raw milk samples in the present study. This discrepancy is likely related to differences in sampling sources. While the cited studies primarily examined raw milk at the farm level or before packaging, the raw milk analyzed in this study was from market-sold products that typically undergo more rigorous hygiene controls and cold-chain preservation before reaching consumers. This difference in sampling stage may explain the low number of *E. coli* in the current findings. Özadam and Özpınar (45) detected *E. coli* in 4% of cheese samples collected in Istanbul, whereas Imre et al. (46) reported its presence in 81.1% of cheeses made from raw milk. The detection of *E. coli* in pasteurized or UHT milk is generally associated with post-processing contamination, whereas the salt used during cheese-making can subsequently suppress bacterial growth. In Romania, Imre et al. (46) used the ISO standard and reported high levels of *E. coli* in raw-milk cheeses. It should also be emphasized that cheese production practices vary widely across regions, which may help explain differences in prevalence rates.

Shiga toxin–producing *E. coli* (STEC) is commonly referred to as EHEC. The most notable STEC serotype is O157: H7, which is frequently associated with severe clinical outcomes and large-scale outbreaks worldwide. Transmission generally occurs through ingestion of contaminated food. Humans typically acquire STEC infection via the oral route, with contaminated food, particularly raw milk and meat products, being identified as the primary sources. It is noteworthy that only a very small infectious dose is necessary to induce disease. Specifically, ingestion of as few as 10–100 STEC O157: H7 bacteria can cause illness in humans (47).

Kholdi et al. (48) reported STEC in 52% of beef samples and identified the O157 serogroup in 3.8% of samples in a study conducted in Iran. Similarly, Ullah et al. (49) examined non-O157 STEC in raw milk and documented a prevalence of 5%. Conversely, Kahraman and Aydin (50), in an investigation in the South Marmara region of Istanbul, did not detect the O157 strain in raw meat samples (including 100 raw beef, 100 raw sheep, 175 raw poultry, and 178 ground beef) nor in meat products such as 138 sausages. Soycan et al. (51) identified STEC in 12% of 220 raw food samples, including beef, chicken, fish, leafy greens, and raw milk, in Kocaeli, a province near Istanbul. This study observed a lower STEC/EHEC detection rate than previously reported by other studies, such as Soycan et al. (51). *E. coli* was most frequently isolated from turkey meat; however, the primary reservoir for the O157 strain is beef, which may account for its absence in these findings. Furthermore, because STEC can be introduced into food products through cross-contamination, adherence to hygienic practices is crucial for influencing STEC prevalence. For comparison, Soycan et al. (51) reported a prevalence of 32.65% in beef and 13.33% in raw milk. The discrepancies between their results and this study’s findings may be due to differences in the media employed and the more extensive serotype analysis conducted by Soycan et al. (51). These observations underscore the importance of conducting more comprehensive investigations into virulence genes and serotype diversity in foodborne *E. coli*.

Antibiotic resistance is a significant global health challenge, driven primarily by the excessive, indiscriminate, and frequent use of antibiotics. Food plays a crucial role in the dissemination of antibiotic resistance. Foodborne bacteria may acquire resistance genes from the environment—particularly from agricultural settings—and subsequently transmit them to humans through the food supply. Furthermore, the widespread use of antibiotics in livestock, for purposes such as disease prevention and growth promotion, further accelerates the development of resistance. Contaminated animal waste containing resistant bacteria and genes contributes to the environmental spread of resistance, thereby establishing a continuous cycle of exposure (52).

Menk-Costa et al. (53) identified *E. coli* in 28% of 150 beef samples collected in Brazil and reported the highest ampicillin resistance; resistance rates were even higher in poultry meat. Similarly, Aydin et al. (17) observed elevated antibiotic resistance in raw chicken meat in Istanbul. Furthermore, Menk-Costa et al. (53) reported resistance rates of 51% to tetracycline and 46% to ciprofloxacin among isolates from poultry, beef, and pork. Conversely, Aydin et al. (17) recorded comparable ciprofloxacin resistance in poultry meat. The consistent rates of ciprofloxacin resistance across various food sources and geographic regions underscore the pervasive and enduring nature of this resistance mechanism. In this study as well, a high rate of ampicillin resistance was observed, and ciprofloxacin resistance was found at a similar rate to that reported by Aydin et al. (17). The fact that Aydin et al. (17) found similar rates in raw chicken meat from the same city indicates the widespread use of ciprofloxacin in food-producing animals in Türkiye and the resulting development of resistance. Brazil, like Türkiye, is an exporter of poultry meat, and the similar prevalence of resistance suggests that the same group of antibiotics is being used. Reports of emerging antibiotic resistance from different countries, as identified in this study, highlight the threat of MDR.

Aworh et al. (41) documented MDR in 83% of *E. coli* isolates from turkey meat in the United States. By contrast, their investigation of chicken meat reported a significantly lower MDR prevalence of 9.5%. However, Menk-Costa et al. (53) and Aydin et al. (17) reported markedly higher MDR rates in chicken products. A notable factor that may help explain this variation is the difference in sample types: Aworh et al. (41) focused exclusively on chicken breast specimens, whereas Aydin et al. (17) reported the highest MDR prevalence in chicken wings. This variability underscores the importance of sample selection in assessing antimicrobial resistance in poultry commodities. Gosling et al. (54) observed that ciprofloxacin-resistant *E. coli* was present in 22.4% of turkey breeding flocks and 60.9% of turkey meat flocks in the United Kingdom, with 87% of the isolates demonstrating MDR. These findings underscore the considerable variability in antibiotic resistance rates, influenced by factors such as flock type (breeding versus meat production), farming practices, and regional conditions where the animals are raised and slaughtered. In this study, MDR was detected in 75.8% of cases. As in other studies, this is a concerning finding from a public health perspective. As reported by Aworh et al. (41), turkey meat has been identified as a high-risk food, similar to chicken meat. Therefore, it is critical to pay close attention to the food hygiene and safety of poultry meat and implement more stringent measures than for red meat to address antibiotic resistance. The data presented by Gosling et al. (54) from the United Kingdom, Aworh et al. (41) from the United States, and this study from Turkey clearly demonstrate that antibiotic resistance is a global problem and that food products carry these antibiotic-resistant bacteria.

Gündoğan and Avcı (55) reported resistance rates in *E. coli* isolates from white cheese, raw milk, and ice cream in Türkiye, including ampicillin (90.5%), tetracycline (66.3%), trimethoprim/sulfamethoxazole (44.2%), chloramphenicol (29.4%), and ciprofloxacin (22.4%). All isolates were susceptible to cefotaxime (55). In Ankara, another province in Türkiye, the study similarly reported high rates of ampicillin resistance, aligning with previous findings. Nonetheless, the most notable distinction was the increased resistance to fluoroquinolones, chloramphenicol, and cefotaxime observed in this research. The approximately decade-long interval between the two studies, alongside variations in the origins of the food samples from the respective provinces, may explain these differences. Conversely, Ombarak et al. (18) reported lower resistance rates in *E. coli* isolates from raw milk and cheese produced from raw milk, including tetracycline (27.5%), ampicillin (18.9%), sulfamethoxazole-trimethoprim (11.3%), cefotaxime (4.5%), ceftazidime (3.6%), chloramphenicol (2.3%), and ciprofloxacin (1.4%), and documented a 50% prevalence of MDR. The findings of Ombarak et al. (18) demonstrate reduced resistance across all antibiotic categories. Conversely, the elevated prevalence of cephalosporin and carbapenem resistance observed in this investigation is particularly concerning. Discrepancies in the types and usage frequencies of antibiotics across geographic regions may account for these variations. Furthermore, chloramphenicol is prohibited in numerous countries for use in food-producing animals, and such regulatory disparities may contribute to the significant international variation in resistance rates (56). Following the development of antibiotic resistance, the phasing out of certain antibiotics in poultry production in some countries, or the reduction in their use due to legal restrictions, has led to a partial decline in certain types of antibiotic resistance (57, 58). As shown in Table 3, the prevalence of MDR has varied across food products and years. Possible reasons include varying rates of *E. coli* isolation across different food sources, differences in antibiotics used in animals, and variations in antibiotic withdrawal periods in poultry and cattle. Notably, resistance to ampicillin was consistently the highest in beef, turkey meat, and raw milk.

ESBL-producing *Enterobacterales* are a major public health concern and require close surveillance. Carbapenems and colistin are often considered last-resort antibiotics for treating infections caused by ESBL-producing strains. However, resistance to these agents can be transferred via mobile genetic elements, facilitating rapid dissemination among bacterial populations. For this reason, carbapenem- and colistin-resistant *Enterobacterales* have been classified by the World Health Organization (WHO) as critical priority pathogens requiring urgent monitoring and control measures (15).

In a study conducted in Türkiye, a total of 173 samples were collected: 136 raw milk samples, 17 bulk tank milk samples, and 20 cheese production samples. A total of 64 isolates were identified as ESBL-producing *Enterobacterales*. Among the isolates, 41% originated from raw milk, 36% from bulk tank milk, and 23% from cheese production processes. Of all isolates, 10.9% were *E. coli*, and 71.43% of these were ESBL producers (24). In a study conducted in the Czech Republic between 2010 and 2013, 263 raw milk samples were collected from 40 farms. Analysis revealed that 30.4% of the isolated *E. coli* strains were resistant to beta-lactam antibiotics. Furthermore, molecular characterization showed that 6.6% of the isolates carried the *bla*_TEM_ gene, while 0.7% harbored the *bla*_CTX-M_ gene (38). Studies have generally reported lower rates of ESBL-producing *E. coli* than those reported in the present study. Variations in prevalence can be attributed to differences in sampling years and geographical factors. Istanbul, as Türkiye’s most populous and most tourist-rich metropolis, receives food products from diverse, often untraceable sources. Given its central role and high population density, the presence of ESBL-producing *E. coli* in this region poses a particularly significant public health risk.

In a study conducted in Finland, turkey meat samples were collected from 82 turkey flocks at a single slaughterhouse, which accounts for nearly all of Finland’s turkey meat production. Each flock contained approximately 3,000–6,000 turkeys, and sampling was performed using one fecal swab per transport crate, with five crates sampled per flock, resulting in a total of 410 crates. The analysis revealed that ESBL/AmpC-producing *E. coli* was isolated in 6.1% of the 82 flocks (59). In a German study, cefotaxime-resistant *E. coli* was isolated from 4.2% of beef samples; molecular analysis detected the *bla*_CTX-M_ gene in 11 isolates and the *bla*_SHV_ gene in 1 isolate. In contrast, 40.1% of turkey meat samples yielded cefotaxime-resistant *E. coli*, and 30.5% of these turkey isolates were confirmed as ESBL/pAmpC producers. Molecular characterization further revealed the presence of *bla*_CTX-M_ in 58 isolates and *bla*_SHV_ in 6 isolates (60). Given the high diversity of ESBL genes, only a limited number are likely to be identified in this study. The phenotypic resistance rate is significantly higher than the rate of detected resistance genes, particularly in poultry. This finding suggests the need for increased vigilance and stricter control measures by authorities. Among the resistance genes, the *bla*_CTX-M_ gene is frequently reported in the literature, while the prevalence of the *bla*_TEM_ gene in Istanbul may be a primary factor contributing to the high levels of ampicillin resistance (17).

While the first set of primers (33) detected a certain number of CTX-M group 1 isolates, a more in-depth PCR analysis of CTX-M (34) revealed a higher proportion of isolates containing CTX-M. Even when targeting the same region, these varying proportions highlight the importance of primer selection in PCR analyses. In this study, different primer sequences were used to screen for and distinguish specific subgroups within the CTX-M family. Rather than assuming a uniform genetic structure, different groups were targeted to identify CTX-M-type broad-spectrum beta-lactamases. Consequently, the use of group-specific primers tailored to the conserved regions of each CTX-M cluster is of critical methodological importance. This approach eliminates cross-reactivity, prevents false-negative screening results, and aids in the more accurate epidemiological characterization of ESBL-producing isolates (61). The difference between the primer set used to detect the first ESBL gene in this study (33) and the set used only for *bla*_CTX-M_ (34) is clear. Therefore, to protect public health, further research into plasmid-derived, transferable resistance genes using advanced molecular techniques is required, and the Ministry of Health should monitor this research. The diversity of transferable resistance genes among *E. coli* isolates from different food types poses a threat to global health, and monitoring systems need to be developed to address this.

In this study, varying degrees of phenotypic co-resistance to ampicillin, ciprofloxacin, tetracycline, and chloramphenicol were observed among ESBL-producing isolates. This MDR can be mechanistically explained by the genetic structure of mobile genetic elements that encode ESBLs. In *Enterobacterales* (particularly *E. coli*), CTX-M genes are typically carried on large, conjugative plasmids (e.g., IncF or IncI1) that function as multi-drug resistance platforms. These plasmids frequently harbour resistance determinants against non-beta-lactam antibiotics, such as *tetA*/*tetB* for tetracycline, *cat* or *floR* for chloramphenicol, and plasmid-mediated quinolone resistance (PMQR) genes (*qnrS* or *aac*(6′) -*Ib-cr*) for ciprofloxacin. Overall, our genotypic screening highlights that the spread of CTX-M variants in the food chain is closely associated with the co-carriage of various resistance genes, and that this situation constitutes a comprehensive MDR problem within the “One Health” framework (62, 63).

A study of 427 raw milk samples from Central Anatolia, Türkiye, reported no carbapenem-resistant *Enterobacterales* or associated resistance genes (64). In South Korea, a separate investigation found that 0.5% of *E. coli* strains isolated from the milk of cows with mastitis between 2012 and 2015 were colistin-resistant, and the *mcr*-1 gene was not detected (65). Furthermore, research in Germany identified six colistin-resistant *E. coli* isolates from 909 fecal samples of beef cattle and one colistin-resistant isolate from 196 bulk tank milk samples (66). In Southern Italy, *E. coli* was detected in 147 of 570 retail meat products, and the *mcr* gene was detected in three samples (67). Carbapenems and colistin, regarded as last-resort treatments, have seen increased use recently, despite colistin’s well-documented nephrotoxic and neurotoxic effects. The present study found no resistance genes associated with either colistin or carbapenems. Notably, the genes analysed were plasmid-derived, aligning with the primary objective of this research: quantifying antibiotic resistance transmissible to humans via the food chain. Although the infrequent use of these antibiotic classes has thus far limited the development of resistance, the detection of resistant strains remains a public health concern. This situation underscores the need for more comprehensive screening protocols and more rigorous measures to address this emerging threat. The relationship between the presence of carbapenem resistance genes and phenotypic resistance—involving gene expression, regulation, enzyme kinetics, and cellular barrier mechanisms—is, by its very nature, a complex phenomenon characterized by interconnected components and distinct dynamics. This situation indicates that resistance genes are maintained as a hidden reservoir within bacterial genomes and mobile genetic elements, creating a potential capacity for rapid activation under antibiotic selection pressure. This study demonstrates that the plasmid-derived resistance genes examined are not associated with the carbapenem resistance observed in the phenotype and that different resistance genes are responsible. While this study specifically examined plasmid-derived resistance genes, the presence of chromosomal-derived resistance genes should not be overlooked.

Although antibiotic resistance is primarily studied in pathogenic bacteria, more in-depth research is needed to fully explain the processes underlying the transfer of these genes. *E. coli* has many commensal strains that play a role in the transfer of antibiotic resistance genes. Although this study did not definitively determine whether the strains were pathogenic, it is clear that *E. coli* strains are more prone than other bacteria to acquiring antibiotic resistance genes (13). It is likely that colistin resistance occurs in both commensal and pathogenic *E. coli* strains, as supported by various studies (19, 68, 69).

Figure 1 shows that when the biofilm-forming capacities of *E. coli* isolates were compared across three culture media, no significant differences were observed (*p* > 0.05). Another study, Aydin et al. (17) reported that 44% of *E. coli* isolates obtained from chicken meat in Istanbul formed biofilms in 1% sucrose TSB. Barilli et al. (70) reported that 25% of retail meat products produced *E. coli* biofilms, as determined by PCR. Vijay et al. (71) observed that 51.8% of *E. coli* strains were moderate biofilm producers in their study, which employed the microplate method in lutein broth. It has been observed that foodborne *E. coli* enhances its resistance to environmental conditions through biofilm production, thereby posing a significant threat to the food industry. The variation in biofilm production across environments indicates the need for control measures. It is imperative to recognize that biofilm-producing *E. coli* strains pose a serious health threat because they can transfer antibiotic resistance. The separation of cells or cell clusters, endotoxin production, increased resistance to the host immune system, and the creation of an environment conducive to the formation of resistant organisms are biofilm processes that can trigger disease. Biofilms also prevent antibiotics from penetrating bacteria, and the activation of specific stress-response genes and efflux pumps inside the biofilm actively expels antimicrobial agents (72). Consequently, cleaning of materials and equipment, as well as the utilization of materials that hinder bacterial adhesion, are as vital for food hygiene as personnel hygiene. The production of biofilms by *E. coli* isolates varies across media and is not merely a phenotypic variation in food safety but rather an indication of adaptive virulence regulation in response to environmental stimuli. This situation means that different surfaces and food residues encountered in food processing environments can unpredictably increase the pathogen’s ability to adhere to surfaces and its tolerance to disinfectants. Increased biofilm production, particularly under low-nutrient or stress-inducing conditions, can lead to persistent reservoirs on equipment surfaces, cross-contamination, and high initial loads prior to thermal processing, despite sanitation protocols. Therefore, differences in biofilm production observed across media should be considered not only as laboratory findings but also as critical parameters in risk assessment studies that model real food matrices and process stresses (17, 73, 74). Since the samples examined in this study are from different years and food products, it is more logical to use these data for the specific food product rather than collectively. This study provides epidemiologically valuable data by presenting the prevalence of *E. coli*, antibiotic resistance profiles, and biofilm production levels across different food products and years.

## 5 Conclusion

Foodborne *E. coli* poses a significant public health concern, driven not only by pathogenic strains but also by the antibiotic resistance genes it harbours. The prevalence of *E. coli* varies by food source, with turkey meat appearing to pose a greater risk than dairy products. Although *E. coli* is often considered a commensal bacterium, its presence in food is an important indicator of fecal contamination, underscoring the need for rigorous hygiene throughout the food supply chain, from farm to table. The escalating resistance of foodborne *E. coli* isolates to critically important antibiotics such as cefotaxime and meropenem underscores the urgency of close monitoring and immediate preventive measures. Specifically, resistance to third-generation cephalosporins and carbapenems warrants thorough investigation, and such efforts should be integrated within the One Health framework to effectively address the interconnected risks to human, animal, and environmental health.

## Data Availability

The original contributions presented in the study are included in the article/supplementary material, further inquiries can be directed to the corresponding author/s.

## Author’s note

This study is based on the Ph.D. thesis of the first author.
